# The Gut Microbial Community of Midas Cichlid Fish in Repeatedly Evolved Limnetic-Benthic Species Pairs

**DOI:** 10.1371/journal.pone.0095027

**Published:** 2014-04-14

**Authors:** Paolo Franchini, Carmelo Fruciano, Tancred Frickey, Julia C. Jones, Axel Meyer

**Affiliations:** 1 Lehrstuhl für Zoologie und Evolutionsbiologie, Department of Biology, University of Konstanz, Konstanz, Germany; 2 Zukunftskolleg, University of Konstanz, Konstanz, Germany; Ecole Normale Supérieure de Lyon, France

## Abstract

Gut bacterial communities are now known to influence a range of fitness related aspects of organisms. But how different the microbial community is in closely related species, and if these differences can be interpreted as adaptive is still unclear. In this study we compared microbial communities in two sets of closely related sympatric crater lake cichlid fish species pairs that show similar adaptations along the limnetic-benthic axis. The gut microbial community composition differs in the species pair inhabiting the older of two crater lakes. One major difference, relative to other fish, is that in these cichlids that live in hypersaline crater lakes, the microbial community is largely made up of Oceanospirillales (52.28%) which are halotolerant or halophilic bacteria. This analysis opens up further avenues to identify candidate symbiotic or co-evolved bacteria playing a role in adaptation to similar diets and life-styles or even have a role in speciation. Future functional and phylosymbiotic analyses might help to address these issues.

## Introduction

We are only now beginning to understand that individual animals are not solely discrete entities, but they in fact host a plethora of symbiotic microbes that can have significant effects [1]–[3]. The realization of the importance of the roles that microbial symbionts play in eukaryotic life is a source for excitement and is leading to a more complete understanding of evolution [4]–[7]. Host-microbiota interactions are essential for many facets of physiology, ranging from metabolic activity to immune homeostasis [8], [9]. In the vertebrate gut, bacteria are known to play important physiological roles that influence metabolic processes, such as the digestion of complex carbohydrates [10] and the regulation of fat storage [11]. It has recently been shown that gut microbiota composition can also influence behavior and gene expression in key brain regions in mice, with motor control and anxiety-like behavior differing between mice with and without their normal gut microbiota [12]. The importance of this microbiome-gut-brain-axis is becoming increasingly evident, and differences in bacterial community composition have been shown to affect emotional, learning and memory behavior, and problem solving abilities in mammals [13]–[15]. Microbes might even play a role in speciation in eukaryotes [6]. The attention of speciation research in eukaryotes is mostly focused on the genetic mechanisms of divergence [16]–[18], and the potential role of symbiosis is often overlooked [6]. Most studies of vertebrate gut communities concentrated on mammals and analyses on fish have been few so far [19].

**Figure 1:**
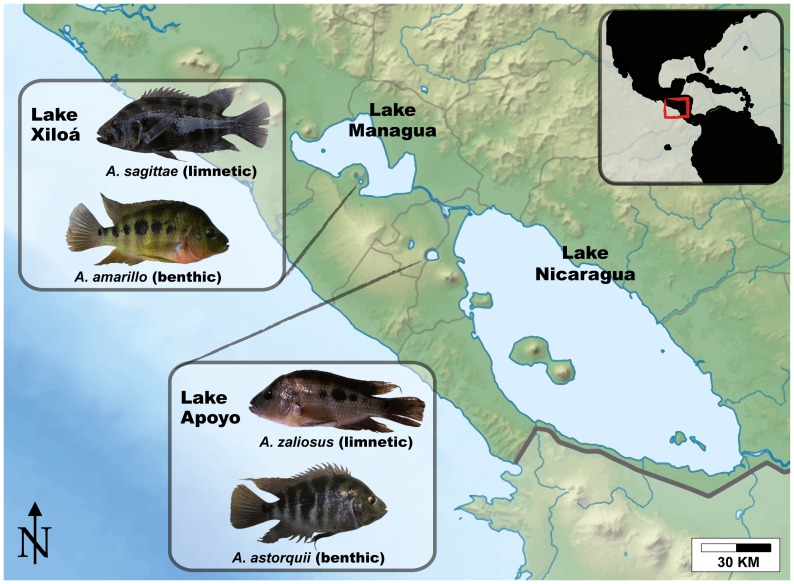
Map of the Nicaraguan main lakes and the two crater lakes, Lake Apoyo and Lake Xiloá. Four typical specimens of *A. astorquii*, *A. zaliosus*, *A. sagittae* and *A. amarillo* are shown.

Cichlid fish are an important model in evolutionary biology as they have repeatedly formed extremely fast adaptive radiations [20], [21]. Midas cichlids from Nicaragua are one of the few empirical cases of sympatric speciation, where specialized open water limnetic and deeper bodied benthic forms have repeatedly and rapidly evolved in different lakes from a common benthic generalist ancestor [22]–[24]. We aimed to characterize the gut microbial communities of Midas cichlid benthic-limnetic species pairs in the two well-studied adaptive radiations of Lakes Apoyo and Xiloá. In each of these lakes, one limnetic species (*Amphilophus zaliosus* and *A. sagittae*, respectively) and multiple benthic species (here we focus on *A. astorquii* and *A. amarillo*) have evolved [25] (Fig. 1).

### Characterizing the Midas cichlid gut microbiota

Next-generation sequencing techniques now permit the fast and cost-effective characterization of microbial communities based on, for example, the hypervariable V4 region of the 16S ribosomal RNA gene [26], [27]. These protocols allow consistent, non-biased characterization of both host-associated and free-living microbial communities. Taking advantage of these developments we sequenced the microbial gut community of lab-reared replicates of benthic-limnetic Midas cichlid species pairs that were collected as juveniles from two crater lakes in Nicaragua and were fed identical diets in the laboratory since then. We aimed to establish a base line for host-microbiota studies of cichlid fish and to ask whether the gut microbiota differed between ecomorphs [28]. If benthic and limnetic species pairs that arose independently, and live in different lakes [29] show parallel differences across lakes, this would speak for an adaptive value of a particular microbiota relating to similarities in their feeding ecology.

## Material and Methods

### Sample collection and preparation

For the present study, we used a total of 12 fish, three of each of the four species: *A. astorquii* (benthic species from Lake Apoyo), *A. zaliosus* (limnetic species from Lake Apoyo), *A. amarillo* (benthic species from Lake Xiloá) and *A. sagittae* (limnetic species from Lake Xiloá). Fish were collected in the wild as fry in 2005 with permission from MARENA and raised under identical conditions at the University of Konstanz animal facility.

The 12 fish chosen for the present study were selected from the above-mentioned wild-caught lab-reared stock based on being as similar as possible in size (standard length 178±15 mm) and being the same sex (only female specimens were used) to remove potentially confounding factors from the analysis. Each fish was sacrificed with an hypothermic treatment and immediately dissected under a hood. A sample of about 200 mg was excised from the gut frontal to the junction between the esophagus and the stomach.

### DNA extraction, amplification and sequencing

DNA extractions were performed with the QIAamp DNA Stool Mini Kit (Qiagen, Valencia, USA) following the manufacturer's protocol. The integrity of each DNA sample was assessed using agarose gel electrophoresis and quantified using a Qubit v2.0 fluorometer (Life Technologies, Darmstadt, Germany). Illumina libraries were prepared following the method described by Caporaso et al. [30] using the NEXTflex 16S V4 Amplicon-Seq Kit (Bioo Scientific, Austin, USA). Briefly, from 50 ng of DNA template for each sample, the bacterial V4 region of the 16S ribosomal gene was amplified using the universal primers 515F and 806R tailed with Illumina barcoded adapters [26] following the PCR conditions recommended by the manufacturer. PCR products were purified using the Agencourt XP Ampure Beads (Beckam Coulter, Inc.) and, subsequently, aspecific DNA fragments were removed using the MinElute Gel Extraction Kit (Qiagen). The quality of the final products was assessed using a Bioanalyzer 2100 (Agilent Technologies, Waldbronn, Germany) and, after their quantification with a Qubit, the samples were pooled in equal proportions and sequenced paired-end in an Illumina MiSeq with 312 cycles (151 cycles for each paired read and 10 cycles for the barcode sequence). To prevent focusing, template building and phasing problems due to the sequencing of “low diversity” libraries such as 16S amplicons, 50% PhiX genome was spiked in the pooled library. All sequences have been deposited in the NCBI's Sequence Read Archive (SRA accession number to be provided upon acceptance).

### Sequence processing

SeqPrep (https://github.com/jstjohn/SeqPrep) was used to remove any reads that were contaminated with Illumina adapters and to merge overlapping paired-end reads into single longer reads covering the full 16S V4 region (254 bp). To avoid the generation of incorrect sequences, the minimum overlapping length was set to 15 bp. Next, the resulting sets of paired- (merged) reads were filtered for quality using CLC Genomics Workbench v6.5 (CLC bio, Aarhus, Denmark). Low quality reads (CLC “limit” set to 0.01) were discarded. To improve taxonomic assignment, only reads representing the full 16S V4 region (252 to 254 bp) were retained for downstream analyses.

### Characterization of the microbial communities of the studied species

For a general characterization of the main microbial taxa found in the four cichlid fish species studied here, we used the Visualization and Analysis of Microbial Population Structures (VAMPS, http://vamps.mbl.edu/) webservice. Next-generation processed reads were clustered into Operational Taxonomic Units (OTUs) using a 97% identity threshold with USEARCH [31] and then information on the most representative taxa were extracted. We also performed a principal coordinate analysis based on the matrix of pairwise Euclidean distances between samples obtained in MG-RAST [32] using the lowest common ancestor method (LCA; [33]) for taxonomic annotation.

### Analysis of the difference in microbial composition between benthic and limnetic fish

To test if benthic and limnetic Midas cichlids are characterized by different microbial communities we used a two-step approach. First, we tested for overall difference in microbial communities between ecomorphs, then we identified the relevant bacterial taxa by performing taxonomical assignment on sequences whose number of reads were different between benthic and limnetic species. For the analysis of the overall difference in microbial communities we performed the permutation test with UniFrac weighted distances [34] implemented in QIIME [30] both comparing benthic and limnetic fish pooled between lakes and performing separate comparisons for *A. astorquii* vs *A. zaliosus* and *A. amarillo* vs *A. sagittae*.

To identify bacterial taxa present differentially in benthic and limnetic fish, we extracted the raw counts of the number of sequences that were assigned to each of the OTUs obtained with the USEARCH clustering algorithm at a 97% similarity threshold. Then, to test which OTUs were differentially represented in each pre-defined group, we compared benthic and limnetic fish both pooled between lakes and within each lake, using Metastats [35]. We then used SGoF+ [36] to control for the false discovery rate. OTUs were deemed significant at the 0.01 false discovery rate using the above-mentioned procedure. Finally, the consensus sequence of each OTU that was statistically different between each group was assigned to the lowest possible taxonomic level using a BLASTn similarity search against the NCBI 16S ribosomal RNA sequence database. In the cases where the taxonomic assignment did not reach the genus level, we also identified the genera with best-hit BLAST scores.

### Ethics statement

The work described here has been conducted according to German law on animal welfare and specifically approved by the Regierungspräsdium Freiburg, Abteilung Landwirtschaft, Ländlicher Raum, Veterinär- und Lebensmittelwesen.

## Results and Discussion

**Figure 2:**
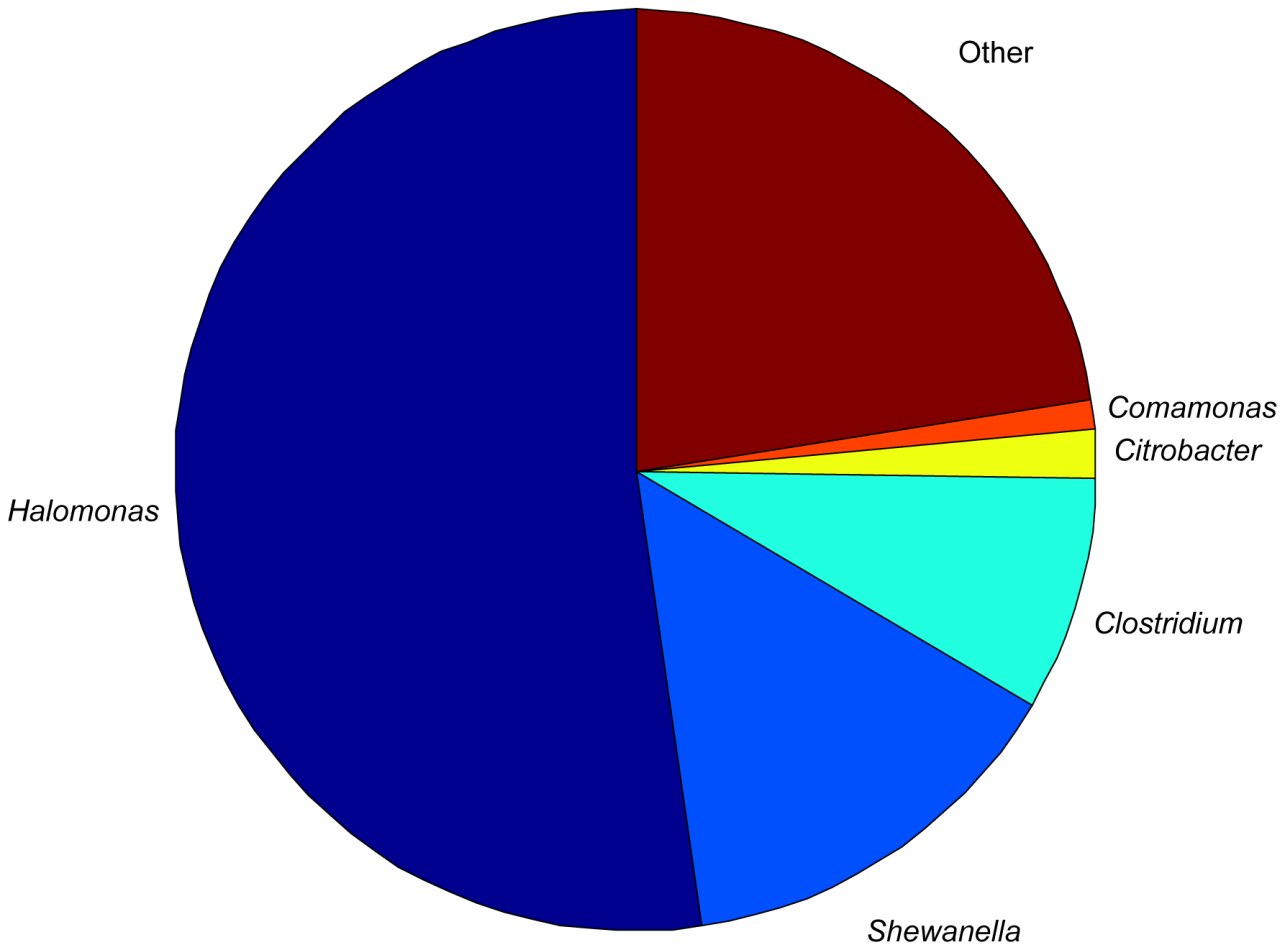
Pie chart showing the abundance of the OTUs with a frequency higher than 1% in the pooled sample. Halomonas, the most abundant OTU, was not reported in a previous survey of teleost gut microbiota.

Massive parallel sequencing provides an unprecedented opportunity to define the bacterial types that are broadly shared among Midas cichlid fish. We analyzed the sequence variation of 3,347,172 complete 16S rRNA V4 regions (an average of 278,931 sequences per individual – standard deviation: 47,399; complete sequencing statistics in Table S1) and identified members of the Gammaproteobacteria class (genus *Halomonas* 52% and also *Shewanella* 14% of total reads) as the most common members of the gut microbiota of adult Midas cichlid fish (see Fig. 2 and Table 1). Even when analyzed at the individual level, the clusters corresponding to these two genera are present in all our samples and constitute a large proportion of their gut microbiota. In fact, Cluster6060 (assigned to the genus *Halomonas*, see also Table 1) ranges between 31.3% (an *A. amarillo* individual) and 69.9% (an *A. zaliosus* individual) of total reads at the individual level. Cluster8707 (assigned to the genus *Shewanella*; Table 1) makes up between 8.4% (in an *A. astorquii* specimen) and 18.8% (in an *A. zaliosus* specimen) of the total reads at the individual level. The same bacterial phyla (predominantly Proteobacteria, but also Firmicutes) were found in gut bacterial communities of other teleost fish in both culture-independent and culture-based surveys [19], [37]–[44]. This commonality of phylotypes suggests that fish harbor bacteria that are typical of the fish gut environment – despite large evolutionary and geographic distances between their fish hosts – rather than reflecting communities from their surrounding environment [19], [45].

**Table 1:** Taxonomic assignment of the best hits for clusters (OTUs) with a frequency higher than 1% in the pooled sample.

Cluster	Phylum	Class	Order	Family	Genus	Best hit(s)	Accession	Abundance (%)
**Cluster6060**	Proteobacteria	Gammaproteobacteria	Oceanospirillales	Halomonadaceae	*Halomonas*	*Halomonas salifodinae* strain BC7	NR_044263.1	52.28428333
						*Halomonas saccharevitans* strain AJ275	NR_044117.1	
						*Halomonas denitrificans* strain M29	NR_042491.1	
						*Halomonas kenyensis* strain AIR-2	NR_043299.1	
						*Halomonas campaniensis* strain 5AG	NR_042157.1	
						*Halomonas desiderata* strain FB2	NR_026274.1	
**Cluster8707**	Proteobacteria	Gammaproteobacteria	Alteromonadales	Shewanellaceae	*Shewanella*	*Shewanella haliotis* strain DW01	NR_044134.1	14.16401667
						*Shewanella algae* strain OK-1	NR_028673.1	
**Cluster8392**	Firmicutes	Clostridia	Clostridiales	Clostridiaceae	*Clostridium*	*Clostridium perfringens* strain 13	NR_074482.1	8.269458333
						*Sarcina maxima* strain DSM 316	NR_026147.1	
						*Sarcina ventriculi* strain DSM 286	NR_026146.1	
**Cluster4535**	Proteobacteria	Gammaproteobacteria	Enterobacteriales	Enterobacteriaceae	*Citrobacter*	*Citrobacter freundii* strain DSM 30039	NR_028894.1	1.730133333
						*Raoultella terrigena* strain 84	NR_037085.1	
						*Citrobacter werkmanii* strain CDC 0876-58	NR_024862.1	
						*Citrobacter murliniae* strain CDC 2970-59	NR_028688.1	
						*Citrobacter braakii* strain 167	NR_028687.1	
						*Citrobacter gillenii* strain CDC	NR_041697.1	
**Cluster8049**	Proteobacteria	Betaproteobacteria	Burkholderiales	Comamonadaceae	*Comamonas*	*Comamonas testosteroni* CNB-2 strain CNB-1	NR_102841.1	1.00715
						*Comamonas testosteroni* strain KS 0043	NR_029161.1	
**Total**								**77.45504167**

We found one major difference in the composition of the microbiota of Midas cichlids guts relative to what has been found in other teleost species [19]: members of the Family Halomonadaceae (Order Oceanospirillales) were the most common bacteria. This order was absent in a recent fish bacteria meta-analysis of teleost gut communities based on 19 different species [19]. Generally this order is reported to be halotolerant or halophilic, i.e. adapted to or living in conditions of high salinity, and aerobically heterotrophic [41], [46], [47], and members of the Halomonadaceae are almost exclusively halophiles [47]. In this context it is worth mentioning that the water in the Nicaraguan crater lakes, and in particular in Lakes Apoyo and Xiloá, is not potable and has a high concentration of dissolved salts when compared to the great lakes, Nicaragua and Managua [48].

Further analyses are required to determine the function of these bacteria, and whether these bacteria perform similar functions to other Proteobacteria found in other fish and vertebrate hosts. Whole-genome comparisons of Bacteroidetes species inhabiting the gut, for example, show that their proteomes have similar functional profiles despite differing in 16S rRNA pairwise identity by as much as 12% [49], [50]. Reciprocal transplants, for example between zebrafish and Midas cichlid gut microbiota, would allow us to examine whether the cichlid gut environment selects or constrains which members of the microbial populations will dominate and persist – as illustrated by Rawls et al. [49] who seeded germ-free zebra fish guts with gut flora from mice.

### Do ecotypes differ in their microbiota communities?

**Figure 3:**
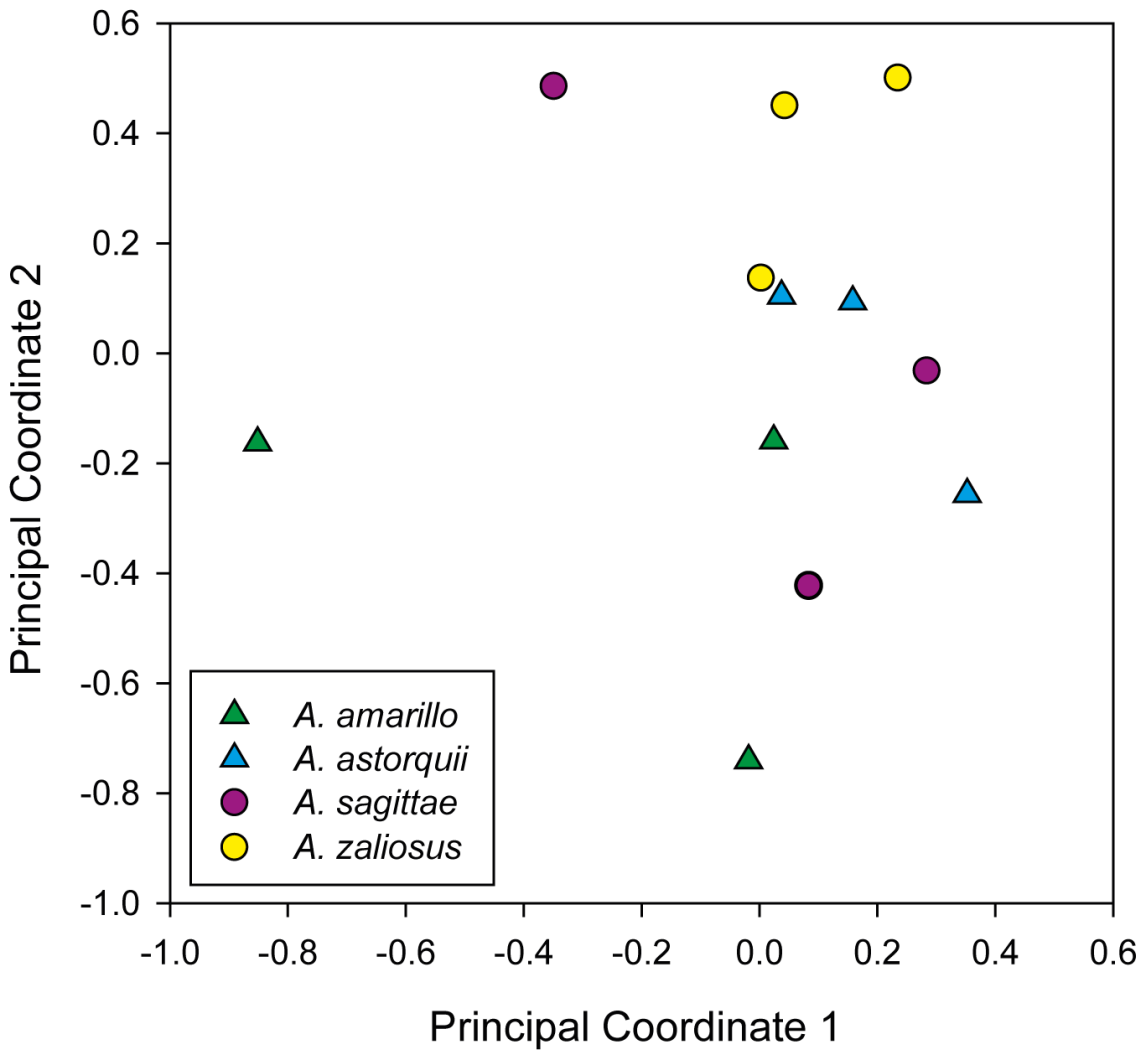
Scatterplot of the scores along the first two principal coordinate axes – explaining 25.72% and 18.67% of variance, respectively – for each sample used in this study.

The benthic-limnetic axis of adaptive differentiation clearly is important in many freshwater systems. Divergent benthic and limnetic forms have been described in many freshwater fishes including sticklebacks [51]–[54], whitefish and other salmonids [55]–[60], perch [61], [62], Neotropical Midas cichlids [63], [64] and African cichlids [65]. The investigation of the genetic basis of this axis of species differentiation and ecological adaptation has been undertaken with several approaches [57], [66]–[69]. In general, studies of species differentiation have centered on the genetics of habitat specificity [6], [70]–[72]. However, recent evidence suggests that bacterial symbionts may play a key role in resource exploitation and specificity, as well [6]. Clearly, there is also a need to analyze the associations that constitute so-called “metaorganisms” [1]. The two-dimensional representation provided by the principal coordinate plot (Fig. 3) shows a certain degree of separation between different species. In particular, within each lake, limnetic species (*A. sagittae* and *A. zaliosus*) show higher scores on the second principal coordinate axis relative to their benthic counterparts.

We explicitly tested whether benthic and limnetic Midas cichlids, which are thought to have arisen rapidly in crater lakes through ecological disruptive selection causing sympatric speciation [23], [24], are characterized by different microbial communities using a permutation test with weighted UniFrac distances – i.e. distances between environmental samples which take into account the phylogenetic relationships among bacterial OTUs within each environmental sample [34]. Because the fish used in this study were reared under common environments, any shifts in microbial community would be expected to represent differences in the core characteristic gut microbes rather than subsequent colonization of the hosts by environmental bacteria.

Our analyses show that even under a common rearing environment, significant differences in the microbiota of limnetic and benthic adult cichlid fish are found (weighted UniFrac test, p = 0.04). Within lakes, the comparisons between benthic and limnetic species produced different results. The species pair inhabiting the older crater Lake Apoyo harbors microbial gut communities that are significantly different (weighted UniFrac distance 0.47, p = 0.03). On the other hand, the gut microbiota of the younger crater Lake Xiloá species pair do not differ significantly (weighted UniFrac distance 0.46, p = 0.08). This result is mirrored by the scatterplot of the scores along the first two principal coordinate axes (Fig. 3) as the clusters of the two species from Lake Apoyo (*A. astorquii* and *A. zaliosus*) do not overlap. On the other hand, the clusters of the two species from Lake Xiloá, while distinct, show a certain degree of overlap. Interestingly, the weighted UniFrac distances between limnetic and benthic fish from different lakes (0.42 and 0.23, respectively) are lower than the distances between ecomorphs within lakes. The lack of differentiation between the gut communities of the Xiloá species might be a consequence of the lake's younger age (maximum of 6100 years;[73]) in comparison to Lake Apoyo (with a maximum of 24000 years;[73]), and therefore also younger age of the Midas cichlid species as bacterial hosts [23], [24], [29]. The disparity observed between parallel species pairs inhabiting different crater lakes may provide a first indication of the rate of differentiation of the core microbial community of fish. Alternatively, the parallel adaptive radiations of these fish in different crater lakes may be affected by different mechanisms, where microbes potentially play a more significant role only in some cases. Further, the results may point to an inherent difference among species in the mechanism by which symbiotic communities assemble within the gut. Future studies should also test the hypothesis that the relationship of microbiomes across host species reflect the hosts' evolutionary history [6], [74].

### How do benthic and limnetic gut communities differ?

To identify more precisely gut bacterial taxa that differ between *A. astorquii* (benthic) and *A. zaliosus* (limnetic) we used a bottom up approach and performed a taxonomical assignment of sequences where read numbers differed between ecomorphs. *A. astorquii* and *A. zaliosus* were different in the relative frequencies of clusters assigned to the genera *Halomonas, Shewanella, Comamonas, Enhydrobacter, Vibrio* (see Table S2). *A. zaliosus* had higher relative sequence counts in most clusters whose abundances were significantly different between the two species. In particular, the clusters identified as belonging to the genera *Shewanella* and *Vibrio* had always higher abundance in *A. zaliosus*. On the other hand, *A. astorquii* had significantly higher abundance for clusters assigned to the genera *Enhydrobacter* and *Comamonas*. Further investigations are required to test for the possible roles of these different cichlid fish gut bacteria, and the general mechanisms of symbiosis and gut community assembly. However, the taxonomic differences identified here represent the first candidate symbionts that might be involved in the maintenance and possibly even the origin of different cichlid species that exploit different ecological niches in individual crater lakes.

## Supporting Information

Table S1 **Sequencing statistics.** For each individual, number of sequences before and after the processing steps are shown.
(XLSX)

Table S2 **For each of the 25 OTUs differentially represented in the comparison between benthic (** ***A. astorquii*** **) and limnetic (** ***A. zaliosus*** **) species of crater Lake Apoyo, sequence similarity search (BLASTn) results are reported.**
(XLSX)
