# Correction: The Gut Microbial Community of Midas Cichlid Fish in Repeatedly Evolved Limnetic-Benthic Species Pairs

**DOI:** 10.1371/journal.pone.0103923

**Published:** 2014-07-23

**Authors:** The PLOS ONE Staff

There is an error in the Materials and Methods section, regarding the authors’ data availability. Under the subheading “DNA extraction, amplification and sequencing” in the final sentence, the correct statement should read as follows: “Raw Illumina sequences were deposited into the NCBI's Sequence Read Archive (SRA) database with accession number: SRA160040.”
